# Sensitivity Improvement of a Surface Plasmon Resonance Sensor Based on Two-Dimensional Materials Hybrid Structure in Visible Region: A Theoretical Study

**DOI:** 10.3390/s20092445

**Published:** 2020-04-25

**Authors:** Zhining Lin, Shujing Chen, Chengyou Lin

**Affiliations:** 1 College of Mathematics and Physics, Beijing University of Chemical Technology, Beijing 100029, China; 2018200906@mail.buct.edu.cn; 2 Beijing Key Laboratory of Materials Utilization of Nonmetallic Minerals and Solid Wastes, National Laboratory of Mineral Materials, School of Materials Science and Technology, China University of Geosciences, Beijing 100083, China; chenshujing@cugb.edu.cn

**Keywords:** surface plasmon resonance, sensor, two-dimensional material, hybrid structure, genetic algorithm

## Abstract

In this paper, we propose a surface plasmon resonance (SPR) sensor based on two-dimensional (2D) materials (graphene, MoS_2_, WS_2_ and WSe_2_) hybrid structure, and theoretically investigate its sensitivity improvement in the visible region. The thickness of metal (Au, Ag or Cu) and the layer number of each 2D material are optimized using genetic algorithms to obtain the highest sensitivity for a specific wavelength of incident light. Then, the sensitivities of proposed SPR sensors with different metal films at various wavelengths are compared. An Ag-based SPR sensor exhibits a higher sensitivity than an Au- or Cu-based one at most wavelengths in the visible region. In addition, the sensitivity of the proposed SPR sensor varies obviously with the wavelength of incident light, and shows a maximum value of 159, 194 or 155°/RIU for Au, Ag or Cu, respectively. It is demonstrated that the sensitivity of the SPR sensor based on 2D materials’ hybrid structure can be further improved by optimizing the wavelength of incident light.

## 1. Introduction

Surface plasmon resonance (SPR) is an optical phenomenon that occurs at the interface between the metal and dielectric. The surface plasma wave (SPW), excited by a specific incident condition, is very sensitive to the change in the refractive index (RI). SPR sensing technology, owing to the merits of no labeling, fast analysist speed and real time [1], is widely applied in various fields, such as biomolecular detection [2], food safety [3], and chemical sensing [4]. A traditional SPR sensor generally employs a Kretschmann configuration, based on attenuated total reflection (ATR) for exciting the SPW, due to its convenience and efficiency. A typical Kretschmann configuration consists of a thin film of metal coated on a high index prism, and a sensing medium touching the metal film [5]. When the propagation constant of the incident light matches with that of the SPW the energy is absorbed, and will form a narrow dip in the reflectance curve that can be used for sensing. 

Sensitivity, defined as the resonance angle or wavelength shift per analyte refractive index unit (RIU), is a significant parameter for an SPR sensor. A few of the schemes based on sensor structure optimization were put forward to enhance the sensitivity. For example, Alleyne at al. created a six-fold enhancement in sensitivity using periodic metallic structures [6]. Kapoor et al. found a SPR sensor with 40 nm ITO and 15 nm ZnO could obtain the sensitivity up to 1620 nm/RIU [7]. Wang et al. achieved 2459.3 nm/RIU by coating WS_2_ on an Au-based SPR sensor, whose sensitivity increased by 26.6% compared to the conventional Au-based sensor [8]. Shukla designed a fiber optic sensor with 40 nm Au and 15 nm ZnO, which realized the sensitivity of 3161 nm/RIU [9].

Two-dimensional (2D) materials have drawn extensive attention to the possibility of enhancing the sensitivity of SPR sensors because of their extraordinary photoelectric properties, unique layered structure and high surface-to-volume ratio. Thus, the 2D materials are prone to surface modification and are beneficial for enhancing the adsorption of biomolecules (such as ss-DNA) [10]. Graphene is a commonly used 2D material for sensitivity improvement in SPR sensors. Sheng et al. raised an SPR sensor with Ag and graphene monolayer and achieved the tunable SPR sensing by changing the thickness of the medium [11]. Verma et al. found that the sensitivity of the sensor with graphene and an air gap can gain 2.35 times higher sensitivity than the normal sensor [12]. Maharana et al. proved that the performance of a gas sensor based on graphene and Ag can be obviously improved, and its imaging sensitivity increases by 340%, 120% or 82% at wavelengths of 653, 800 or 1000 nm respectively [13]. The maximum sensitivity of a fiber biosensor with Cu/graphene construction proposed by Rifat et al. exhibited up to 2000 nm/RIU for wavelength interrogation [14]. Apart from graphene, other new 2D materials, such as molybdenum disulfide (MoS_2_) [15], tungsten disulfide (WS_2_) [15], and tungsten diselenide (WSe_2_) [16], have also been demonstrated as options for the sensitivity enhancement of an SPR sensor. Considering the good properties of 2D materials for sensitivity improvement, the hybrid structure based on multiple 2D materials has also received widespread attention. Rahman et al. dished an Au/MoS_2_/graphene-based biosensor, which showed a sensitivity of 87.8°/RIU [17]. Kushwaha et al. projected a biosensor with an SF10 prism/zinc oxide/Au/MoS_2_/graphene hybrid structure and attained the sensitivity of 101.58°/RIU [18]. Wu et al. proposed a MoS_2_/Al/MoS_2_/graphene hybrid structure and the maximum sensitivity reached 190.83°/RIU [19]. Compared to the common Au-based SPR sensor, more than two times sensitivity enhancement was achieved by the sensor with a 10 layer BP/monolayer WS_2_ heterostructure [20].

In an SPR sensor based on a hybrid structure, the sensitivity optimization becomes difficult due to its complicated construction, which makes the traditional optimization based on manual method inefficient. Genetic algorithm (GA) is a powerful and efficient global optimization method, which mimics biological evolution and can achieve the optimization of multi-parameters and multi-objectives at the same time. At present, GA is widely used in the performance optimization of SPR sensors. Guo et al. reported the sensitivity enhancement of a SPR sensor based on 2D materials (Silver/BP/graphene/MoS_2_/WS_2_/MoSe_2_/WSe_2_) optimized using GA at 633 nm [21]. In our previous research work, an optimization method based on GA with constraint conditions was proposed, and used to design high-performance Au/Ag/dielectric/graphene [22] and Ag/TiO_2_/graphene-based SPR biosensors [23]. Bahrami et al. designed an improved waveguide resonance sensor based on GA to enhance sensitivity. They discovered that refractive index resolution improved by six times more than a traditional one [24]. A gold nanostructure based SPR biosensor based on microgenetic algorithms was presented by Fu et al. for enhancing detection sensitivity [25].

Besides the structure of a sensor, the wavelength of incident light is another key parameter that determines the performance of a SPR sensor. In recent years, the method of improving sensitivity by optimizing the wavelength of incident light has attracted more and more attention. Jha et al. studied the performance of an SPR sensor with an Ag/Au bimetallic alloy in the infrared wavelength region, and demonstrated that the usage of lager Au or Ag nanoparticles, and the larger wavelength (far-infrared region), can achieve a better sensor performance [26]. Xu et al. investigated a sandwich-like (graphene/Al/graphene) structure based SPR sensor, and found that its sensitivity was inversely proportional to wavelength [27]. Aray et al. improved the sensor sensitivity based on indium molybdenum oxide film, using a tunable wavelength [28]. In our previous paper, the sensitivities of graphene-based SPR biosensors with Au, Ag and Cu were analyzed and compared in the visible region, and the sensitivity of three different metal-based sensors with a monolayer graphene all displayed a maximum value with varying wavelengths of the incident light [29].

In this paper, we study the sensitivity improvement of an SPR sensor based on a graphene/MoS_2_/WS_2_/WSe_2_ hybrid structure in the visible region. To explore the maximum sensitivity of the proposed sensor at a specific wavelength and figure out which 2D materials are helpful for improving its sensitivity, the thickness of the metal film and the layer number of each 2D material in the sensor are optimized using GA. Then, the sensitivity of the proposed sensor that utilizes different metals (Au, Ag, Cu) at the different wavelengths in the visible region has been compared. Furthermore, the performances of proposed SPR sensors are investigated, and the origin of sensitivity improvement is determined.

## 2. Materials and Methods

### 2.1. Structure of Proposed SPR Sensor

**Figure 1:**
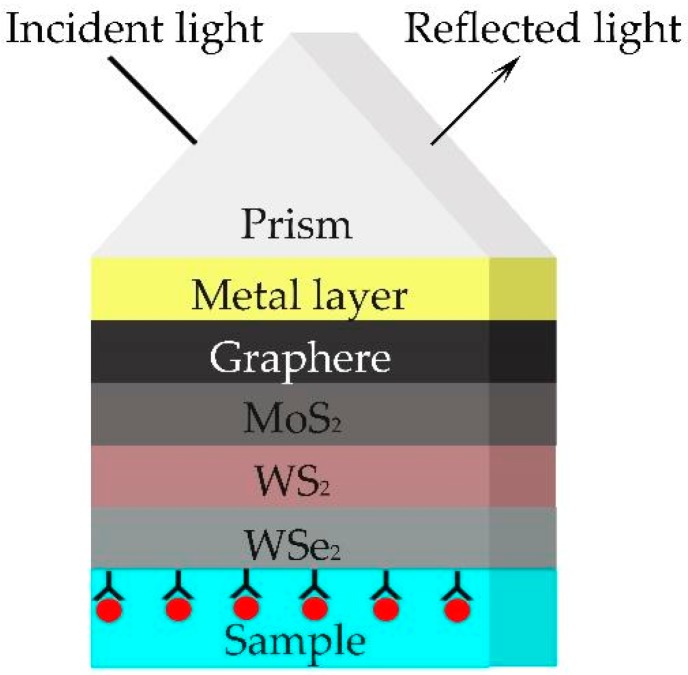
Structure of the proposed surface plasmon residence (SPR) sensor.

The proposed SPR sensor with a prism/metal/graphene/MoS_2_/WS_2_/WSe_2_ structure is shown in Figure 1. In the sensor, we choose SF11 glass as the coupling prism, then use a metal (Au, Ag or Cu) layer covering the prism to excite the SPR. Then, the 2D materials’ hybrid structure (graphene, MoS_2_, WS_2_, WSe_2_) is coated on the metal film. In the present study, the proposed sensor uses a Kretschmann configuration based on angular interrogation, and the sensitivity of the proposed SPR sensor is investigated in the visible region (400–800 nm).

The refractive index of *SF*11 glass can be calculated by the following formula,

(1) $$n_{\mathrm{SF}11}=\sqrt{1+\frac{1.73759695\lambda^{2}}{\lambda^{2}-0.013188707}+\frac{0.313747346\lambda^{2}}{\lambda^{2}-0.0623068142}+\frac{1.89878101\lambda^{2}}{\lambda^{2}-155.23629}},$$

**Figure 2:**
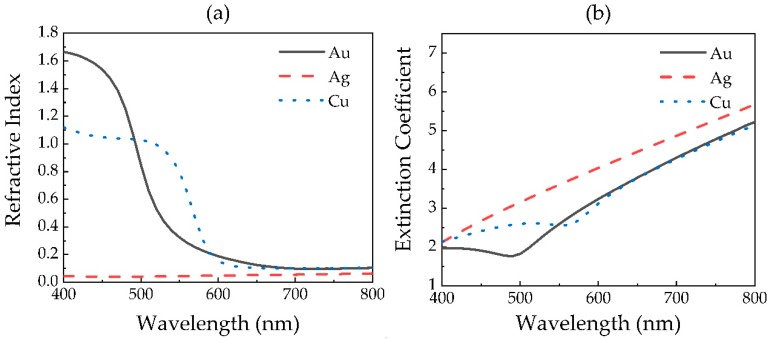
(**a**) Refractive index and (**b**) extinction coefficient of metals in the visible region.

Due to the dispersion of metals in the visible region, the refractive index and extinction coefficient of metals are distinctive at different wavelengths. The refractive index and extinction coefficients of used metals in this paper are taken from [30], shown in Figure 2. It seems that the refractive index of Ag is lower, but its extinction coefficient is higher than that of Au and Cu.

**Figure 3:**
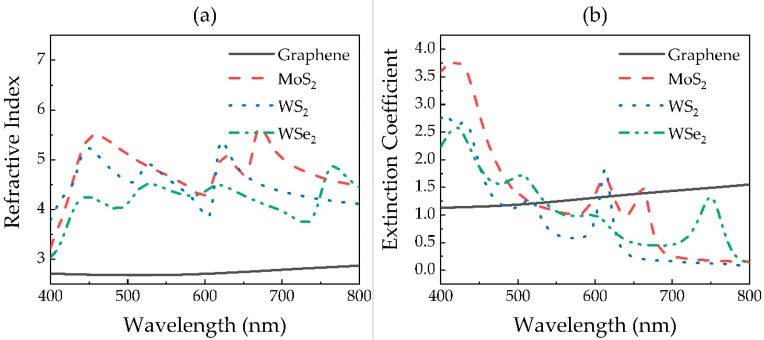
(**a**) Refractive index and (**b**) extinction coefficient of 2D materials in the visible region.

In addition, 2D materials also show obvious dispersion in the visible region, which is shown in Figure 3. All data of refractive index and extinction coefficient originate from [31,32]. In addition, the thickness of the monolayer of each 2D material is exhibited in Table 1 [33].

The sensing medium, which is assumed to be water with ss-DNA biomolecules for our simulations, keeps in contact with the 2D materials. These 2D materials, which work as the biomolecular recognition element, can adsorb the biomolecules on its surface to form an additional ss-DNA layer whose thickness is around 100 nm, and produce a local increase in the refractive index (Δ*n* = 0.005) at the surface of SPR sensor [34]. 

### 2.2. Transfer Matrix Method

The proposed SPR sensor can be regarded as a multilayer structure, and its reflectivity could be calculated by the well-known transfer matrix method as follows

(2) $$M=\left[\begin{array}{cc} A & B\\ C & D \end{array}\right]=\underset{j=1}{\overset{N}{\Pi }}\left[\begin{array}{cc} {\cos\delta }_{j} & {\mathrm{isin}\delta }_{j}/\eta_{j}\\ {i\eta }_{j}{\sin\delta }_{j} & {\cos\delta }_{j} \end{array}\right],$$

(3) $$R=\frac{\eta_{0}\left({A+B\eta }_{N+1}\right){-C-D\eta }_{N+1}}{\eta_{0}\left({A+B\eta }_{N+1}\right){+C-D\eta }_{N+1}},$$

(4) $${R=r\times r}^{*},$$

*M* is the characteristic matrix of the multilayer. *δ_j_ = 2πn_j_d_j_*cos*θ_j_*, in which *n_j_* and *d_j_* are the refractive index and the thickness of each layer in the SPR sensor, and *θ_j_* is the angle of light travelling in each layer. *η_j_ = n_j_*/cos*θ_j_* denotes the optical admittance of each layer for the p-polarized light. *η*_0_ and *η_N_*_+1_ are representative of the optical admittance for incident media (SF11 prism) and emergent media (water). 

The sensitivity (*S*) defines as the ratio of the change in value of the resonance angle to the change in value of refractive index of the analyte, and can be expressed by

(5) $$S=\frac{\Delta \theta_{\mathrm{res}}}{\Delta n},$$

where Δ*θ*_res_ is the offset of resonance angle, and Δ*n* stands for the change in the refractive index of the analyte.

### 2.3. Genetic Algorithm

In order to achieve high sensitivity by optimizing the thickness of metal film and the layer number of each of the 2D materials, a merit function (MF) with a constraint condition is used in GA,

(6) $$\mathrm{MF}=\{\begin{array}{c} {S,\;\theta }_{1}{>\theta }_{0}\\ 0,\;\mathrm{Others} \end{array}.$$

*θ*_0_ and *θ*_1_ are resonance angles before and after ss-DNA addition. The constraint condition of *θ*_1_ > *θ*_0_ in MF is used to avoid a negative shift, which may lead to fake high-sensitivity.

In order to ensure the GA finds the optimal parameters that realize high sensitivity, the population number and the genetic generation in GA are set to 300 and 500, respectively. Besides this, the values of crossover proportion and mutation proportion are both 0.7 to achieve a fast convergence speed. In GA, the optimization range of metal thickness is set to be from 0 to 60 nm, while the range in numbers of layers of 2D materials is set to be from 0 to 15. One thing that should be stressed here is that the optimal 2D materials’ hybrid structure for sensitivity improvement is not always the same for different wavelengths, because the optimized layer number of a certain 2D material may be zero, which means the present type of 2D material is of no use for sensitivity improvement in the proposed SPR sensor, which should be discarded.

## 3. Results

**Figure 4:**
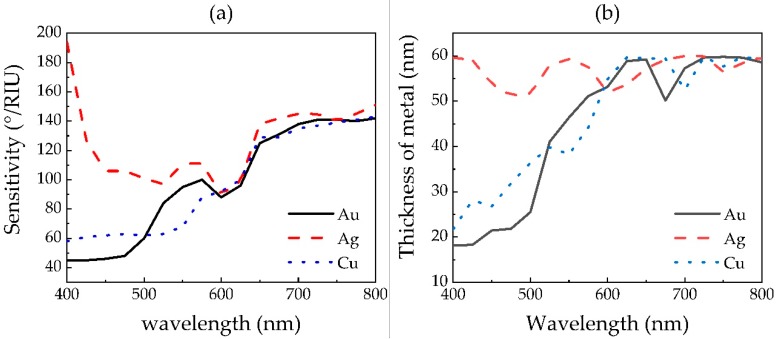
The (**a**) sensitivity and (**b**) metal thickness of optimized 2D materials hybrid structure based SPR sensors with respect to the wavelength of incident light.

First of all, we use the GA to optimize the thickness of the metal and the layer number of each 2D material in the proposed SPR sensors in the visible region. The curve of optimal sensitivity of the proposed SPR sensor with Au, Ag or Cu film, varying with the wavelength changing from 400 to 800 nm, is shown in Figure 4a.

In Figure 4a, the sensitivity is distinct for the optimized SPR sensor with different metal films, originating from the different refractive index and extinction coefficient of various metals, which also causes different optimal thicknesses of metal, as shown in Figure 4b. The sensitivity of Ag-based sensors decreases first and then increases, while the sensitivities of Au- and Cu-based SPR sensors both show an upward trend with increasing wavelength. The sensitivity of the Ag-based sensor is higher than the other metal-based ones at most wavelengths in the considered region, which can be attributed to its lower refractive index and higher extinction coefficient. The maximum sensitivity of an Au-, Ag- or Cu-based SPR sensor is 159, 194 or 155°/RIU. 

**Figure 5:**
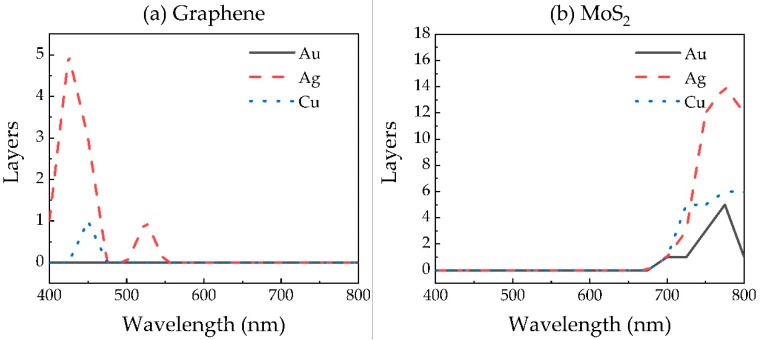

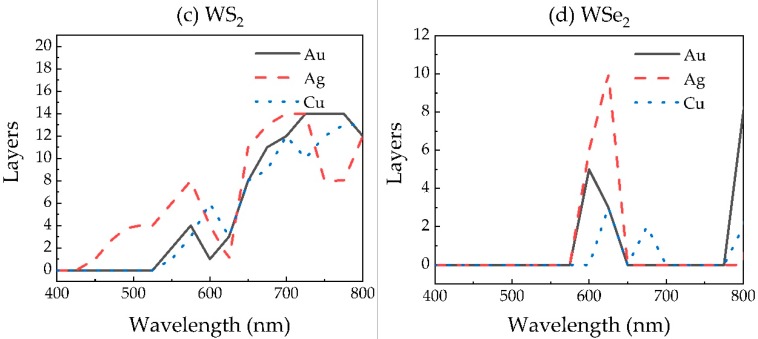
The optimal layer number of (**a**) Graphene, (**b**) MoS_2_, (**c**) WS_2_ and (**d**) WSe_2_ in 2D materials hybrid structure.

The optimal number of layers of each of the 2D materials in a graphene/MoS_2_/WS_2_/WSe_2_ hybrid structure also exhibits a different value for the sensors with different metals, or at different wavelengths, as shown in Figure 5. In addition, it seems that not all 2D materials are needed for sensitivity improvement under a specific wavelength, because many values of the layer number are zero for the proposed sensor. This can be easily understood because each 2D material has its fixed refractive index and extinction coefficient at a specific wavelength, and could affect the sensitivity independently. During the optimization, GA only picks up the 2D materials that increase the merit function (sensitivity). In that case, the materials that have an adverse effect on sensitivity optimization will be discarded. Using this method, we can realize the intelligent selection of 2D materials in the hybrid structure for sensitivity improvement, instead of the tedious manual screening of materials one by one.

**Figure 6:**
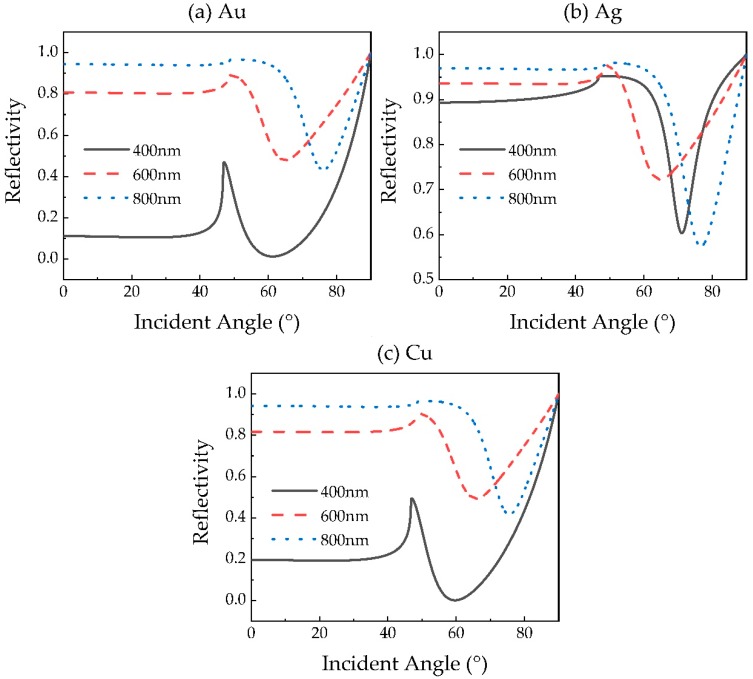
Variation of reflectivity of genetic algorithm (GA)-optimized (**a**) Au-, (**b**) Ag- and (**c**) Cu-based SPR sensors with the incident angle at the wavelengths of 400, 600 and 800 nm.

In order to further study the performances of the SPR sensors with optimized structures, we plot the reflectance curves of the sensors at the wavelengths of 400, 600 and 800 nm, as shown in Figure 6. The parameters of those optimized sensors are listed in Table 2. The letters K, L, M and N in Table 2 are representative of the layer number of graphene, MoS_2_, WS_2_ and WSe_2_, respectively.

As shown in Figure 6, both the resonance angle *θ*_res_ and the sensitivity *S* increase with the wavelength for Au- and Cu-based sensors. Besides this, we find that the SPR sensor exhibiting the higher sensitivity does not necessarily show smaller reflectivity at resonance angle *R*_res_. However, *R*_res_ at 600 nm is always higher than at other wavelengths. At the same time, in conjunction with Table 2, we discovered that the optimized hybrid structure of the sensor with different metals, or at different wavelengths, exhibits a distinct combination of 2D materials. This indicates that the optimal 2D materials’ hybrid structure of the sensor is related to not only the wavelength, but also the metal used in the SPR sensor. It is interesting to see that when the wavelength is lower than 600 nm, all sensor structures do not need MoS_2_, which may be to blame for the high extinction coefficient of MoS_2_ at 400 and 600 nm, as shown in Figure 3b.

**Figure 7:**
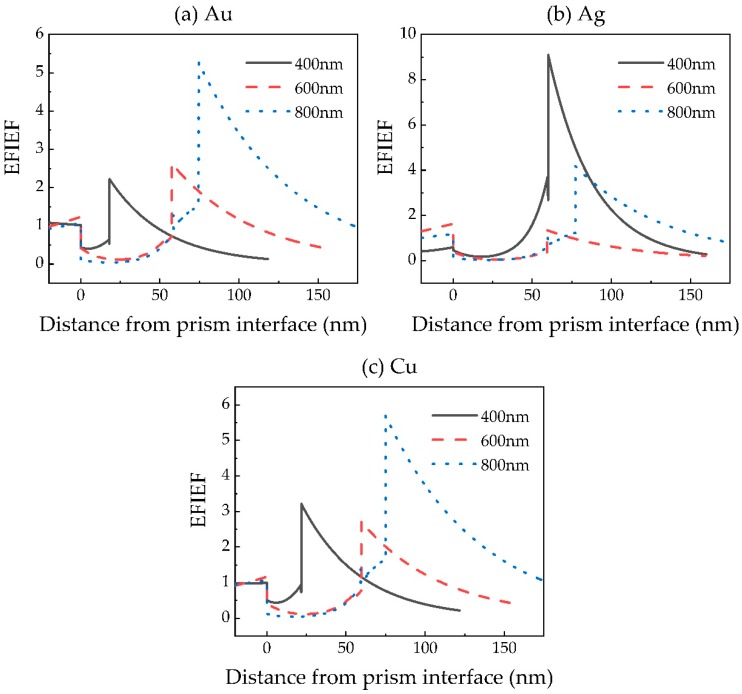
Electric field intensity enhancement factors (EFIEF) of GA-optimized (**a**) Au-, (**b**) Ag- and (**c**) Cu-based SPR sensors.

To study the origin of the sensitivity improvement in 2D material-based SPR sensors, we calculate the electric field intensity enhancement factor (EFIEF) of the proposed sensors using the method described by Shalabney and Abdulhalim [35], as shown in Figure 7. By comparing the sensitivity and the EFIEF of these sensors at various wavelengths, we found that the case which exhibits a larger value of maximum EFIEF always shows higher sensitivity. For example, for an Au-based SPR sensor, the maximum EFIEF is 2.22, 2.63 and 5.27 while the sensitivity is 45, 88 and 142°/RIU at wavelengths 400, 600 and 800 nm. Meanwhile, the SPR sensor with different metals also fulfills this rule. The results indicate that the sensitivity improvement in the proposed sensor is attributed to the electric field intensity enhancement.

Further, a comparison of sensitivity is made based on the other, similar works on 2D material-based SPR sensors, and tabulated in Table 3. From Table 3, it is clear that the proposed SPR sensor can provide a significantly higher sensitivity compared with previously published SPR sensor schemes.

## 4. Conclusions

In summary, we proposed an SPR sensor based on a 2D materials hybrid structure, and investigated the sensitivity improvement of the proposed SPR sensor in the visible region. We achieved the simultaneous optimizations of the thickness of metal (Au, Ag or Cu) and the layer number of 2D materials (graphene, MoS_2_, WS_2_ and WSe_2_) by genetic algorithm, and obtained high sensitivity in the visible region. The results indicate that the sensitivity of the proposed SPR sensor varies obviously with the wavelength of incident light, and shows a maximum value of 159, 194 or 155°/RIU for Au, Ag or Cu respectively. In addition, an Ag-based SPR sensor exhibits a higher sensitivity than an Au- or Cu-based sensor at most wavelengths in the visible region. Furthermore, the optimal number of layers of each 2D material in graphene/MoS_2_/WS_2_/WSe_2_ hybrid structure also exhibits different values for the sensor with a different metal or at a different wavelength. By comparing the sensitivity and the electric field intensity of the optimized sensors at various wavelengths, we demonstrated that the sensitivity improvement in the proposed sensor is attributed to the electric field intensity enhancement. This research not only demonstrates the sensitivity enhancement of 2D material-based SPR sensors by optimizing the wavelength of incident light, but also provides a method that can realize the intelligent selection of 2D materials in a hybrid structure for sensitivity improvement.

## Figures and Tables

**Table 1:** The thickness of 2D material monolayer.

2D Materials	Thickness of Monolayer (nm)
Graphene	0.34
MoS_2_	0.65
WS_2_	0.8
WSe_2_	0.7

**Table 2:** The performance parameters of GA-optimized SPR sensors.

Metal	Wavelength (nm)	Thickness (nm)	K	L	M	N	*R*_res_	*θ*_res_	*S*
Au	400	18.14	0	0	0	0	0.013	61.26	45
	600	53.18	0	0	1	5	0.479	64.89	88
	800	58.58	0	1	12	8	0.433	76.07	142
Ag	400	59.63	1	0	0	0	0.603	71.09	194
	600	51.92	0	0	4	6	0.723	64.76	90
	800	59.78	0	12	12	0	0.574	76.65	151
Cu	400	21.72	0	0	0	0	0.001	59.66	58
	600	54.86	0	0	6	0	0.493	65.82	92
	800	59.20	0	6	13	2	0.417	75.56	143

**Table 3:** Performance comparison of different reported SPR sensors.

Reference	Wave Length (nm)	Configuration	Layer Number of 2D Materials				Sensitivity (°/RIU)
			Graphene	MoS_2_	WS_2_	WSe_2_	
[1]	632.8	BK7/ZnO/Ag/Au/graphene	l	—	—	—	66
[15]	633	SF11/Au/graphene	1	—	—	—	71
[17]	633	SF10/Au/Graphene/MoS_2_	1	2	—	—	89.29
[34]	633	SF10/Au/graphene	1	—	—	—	53.2
[36]	632.8	SF11/Ag/MoS_2_/graphene	1	5	—	—	73.5
[37]	632.8	2S2G /Au/graphene	6	—	—	—	46
[38]	633	SF10/Au/ MoS_2_		6	—	—	75.34
This paper	400	SF11/Ag/graphene	1	—	—	—	194
This paper	600	SF11/Cu/WSe_2_	—	—	—	6	92
This paper	800	SF11/Au/MoS_2_/WS_2_/WSe_2_	—	1	12	8	142
